# 954. Resources Needed by Critical Access Hospitals to Address Identified Infection Prevention and Control Program Gaps

**DOI:** 10.1093/ofid/ofab466.1149

**Published:** 2021-12-04

**Authors:** M Salman Ashraf, Mounica Soma, Jody Scebold, Angela Vasa, Kate Tyner, Sue Beach, Satya Kumar Lalam, Teresa Ann Fitzgerald

**Affiliations:** 1 University of Nebraska Medical Center, Omaha, NE; 2 Nebraska Medicine, Omaha, NE

## Abstract

**Background:**

Critical Access Hospitals (CAH) may face challenges with limited resources in their infection prevention and control (IPC) program. As part of the Project Firstline collaborative, the University of Nebraska Medical Center and its clinical partner Nebraska Medicine sought to identify needs and develop resources to mitigate IPC program gaps in small and rural hospitals, including CAHs. Since, little is known about the resources needed by CAHs to strengthen their IPC program, a needs assessment survey was deployed to Federal Emergency Management Agency Region VII CAHs.

**Methods:**

A 49-question Research Electronic Data Capture (REDCap) survey was distributed via email to infection preventionists in Region VII CAHs. The survey had 4 sections with questions focused on IPC program infrastructure, competency-based training, audit and feedback, and identification and isolation of high-risk pathogens/serious communicable diseases. An IPC practice score was assigned to each CAH by totaling “yes” responses. A “no” or “not sure” response was considered an IPC gap. Respondents who selected “no” were asked to identify resources that would assist in mitigating identified gaps. Descriptive analyses evaluated frequency of gaps and most cited resources. Welch t-test was used to study differences in IPC practice score between states.

**Results:**

50 CAHs (33 in NE, 16 in IA and 1 in KS) and 1 small NE hospital (not licensed as CAH but included in the analyses as CAH) participated in the survey. Majority (n=38) responded to all sections with IPC scores ranging from 13 to 48. There was no significant difference between IPC practice scores of CAHs in NE and IA (average score 33 vs 36; p = 0.38). Specific IPC practice gaps present in > 50% of CAHs were related to audit and feedback practices (Table 1). Additional gaps included lack of drug diversion program, absence of input from IPC team prior to purchasing equipment and failure to conduct risk assessment for the laboratory. Most CAHs cited a standardized audit tool and staff training materials as much needed resources (Table 1).

Table 1. Needs/Resources for the identified Infection Prevention and Control Gaps.

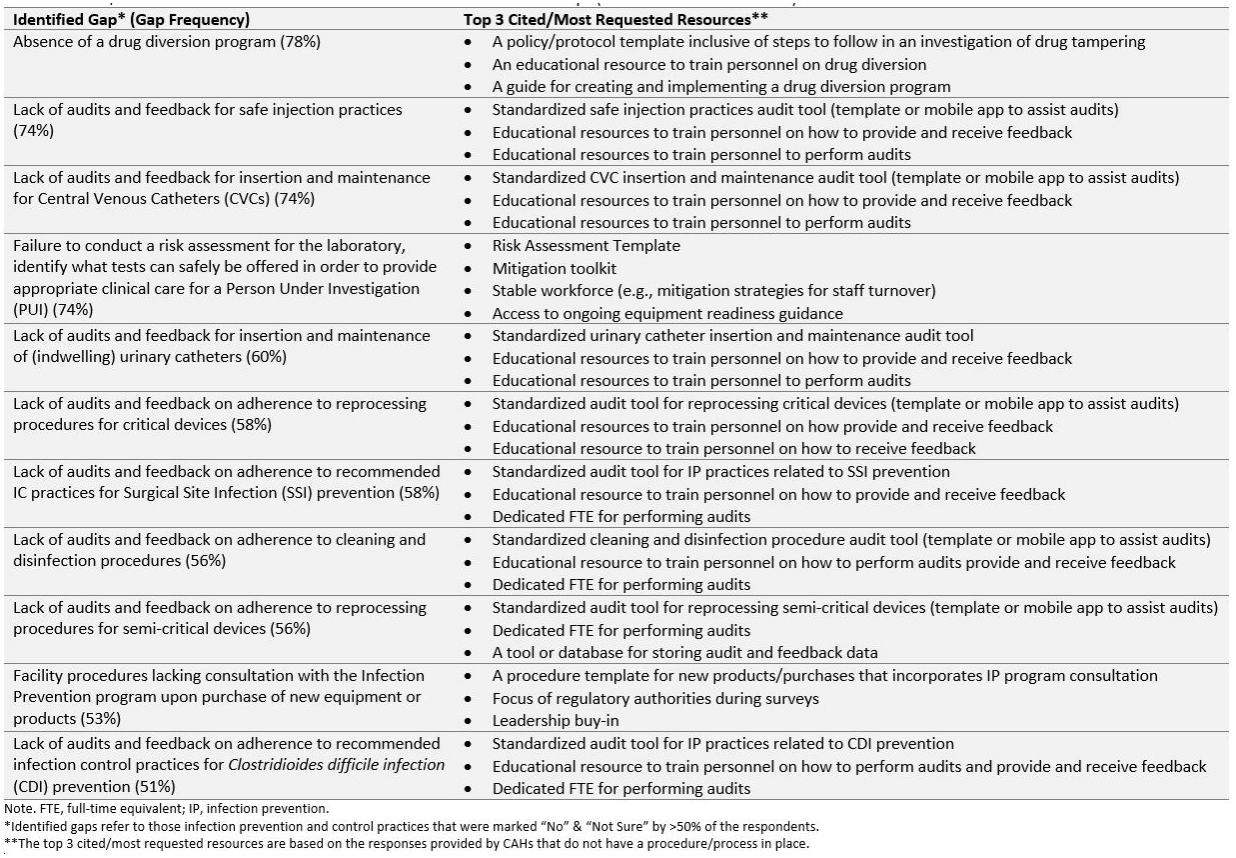

**Conclusion:**

Major IPC gaps exist in CAHs with many of them related to implementing audit and feedback practices that are an essential component of a successful IPC program. Focus should be directed on developing resources to mitigate identified IPC gaps.

**Disclosures:**

**M. Salman Ashraf, MBBS**, **Merck & Co. Inc** (Grant/Research Support, I have recieved grant funding for an investigator initiated research project from Merck & Con. Inc. However, I do not see any direct conflict of interest related to the submitted abstract)

